# An Explainable AI Approach for the Rapid Diagnosis of COVID-19 Using Ensemble Learning Algorithms

**DOI:** 10.3389/fpubh.2022.874455

**Published:** 2022-06-21

**Authors:** Houwu Gong, Miye Wang, Hanxue Zhang, Md Fazla Elahe, Min Jin

**Affiliations:** ^1^Department of Software Engineering, College of Computer Science and Electronic Engineering, Hunan University, Changsha, China; ^2^Academy of Military Sciences, Beijing, China; ^3^Engineering Research Center of Medical Information Technology, Ministry of Education, West China Hospital, Chengdu, China; ^4^Information Center, West China Hospital, Chengdu, China

**Keywords:** artificial intelligence, ensemble learning, explainable, disease prediction, COVID-19

## Abstract

**Background:**

Artificial intelligence-based disease prediction models have a greater potential to screen COVID-19 patients than conventional methods. However, their application has been restricted because of their underlying black-box nature.

**Objective:**

To addressed this issue, an explainable artificial intelligence (XAI) approach was developed to screen patients for COVID-19.

**Methods:**

A retrospective study consisting of 1,737 participants (759 COVID-19 patients and 978 controls) admitted to San Raphael Hospital (OSR) from February to May 2020 was used to construct a diagnosis model. Finally, 32 key blood test indices from 1,374 participants were used for screening patients for COVID-19. Four ensemble learning algorithms were used: random forest (RF), adaptive boosting (AdaBoost), gradient boosting decision tree (GBDT), and extreme gradient boosting (XGBoost). Feature importance from the perspective of the clinical domain and visualized interpretations were illustrated by using local interpretable model-agnostic explanations (LIME) plots.

**Results:**

The GBDT model [area under the curve (AUC): 86.4%; 95% confidence interval (CI) 0.821–0.907] outperformed the RF model (AUC: 85.7%; 95% CI 0.813–0.902), AdaBoost model (AUC: 85.4%; 95% CI 0.810–0.899), and XGBoost model (AUC: 84.9%; 95% CI 0.803–0.894) in distinguishing patients with COVID-19 from those without. The cumulative feature importance of lactate dehydrogenase, white blood cells, and eosinophil counts was 0.145, 0.130, and 0.128, respectively.

**Conclusions:**

Ensemble machining learning (ML) approaches, mainly GBDT and LIME plots, are efficient for screening patients with COVID-19 and might serve as a potential tool in the auxiliary diagnosis of COVID-19. Patients with higher WBC count, higher LDH level, or higher EOT count, were more likely to have COVID-19.

## Introduction

Coronavirus disease 2019 (COVID-19, also called novel coronavirus pneumonia) is characterized by fever, cough, and shortness of breath. COVID-19 spreads rapidly due to its highly infectious nature, and caused huge manpower and material resources losses (1, 2). Early detection, diagnosis, isolation, and treatment are keys to improving the cure and survival rates of COVID-19 patients. To respond to this unprecedented pandemic emergency, early identification of infected patients is very important. Infection with severe acute respiratory syndrome coronavirus 2 (SARS-CoV-2), the virus that causes COVID-19 is typically identified with molecular detection using reverse transcriptase PCR (RT–PCR) as the gold standard (3). However, the test process is time-consuming (no <4 h under ideal conditions) and requires the use of special equipment and reagents and specialized and trained personnel for sample collection. Furthermore, the high cost and slow processing speed of RT-PCR make it less feasible for massive population screening in remote areas or backward countries (4). The development of artificial intelligence (AI) technology has made the mining of medical information and the development of disease prediction models for assisting doctors in disease prediction or diagnosis a popular research subject.

To improve the ability to diagnose COVID-19 and curb the spread of the pandemic, the data science community has proposed several machine learning (ML) models, most of which are based on computed tomography (CT) scans or chest X-rays (5–9). Although promising results have been reported, some concerns have been raised about these efforts, especially the chest X-ray-based solutions, regarding the high incidence of false negative results (10). Additionally, while the CT imaging method is accurate, it is costly and time-consuming and requires specialized equipment. As a result, methods based on this imaging technology are inappropriate for screening. Although various clinical studies (11–15) have emphasized the usefulness of blood test-based diagnoses in providing an effective and low-cost alternative for the early detection of COVID-19, relatively few ML models are based on hematological parameters.

The primary goal of medicine in the 21st century has switched from disease prevention and treatment to health maintenance, and the medical mode has changed from a simple disease treatment mode to the so-called “4P” medical mode: prevention, prediction, personalization, and participation (16). To address issues regarding medical complexity, the methodological system of clinical research is also constantly improving. A disease prediction model is a statistical evaluation method based on disease risk factors that divides scores according to the degree of influence of the underlying factor and calculates the probability of a certain event in the future by a mathematical formula (17). These disease prediction models can enable medical staff to implement targeted intervention measures for patients with different risk probabilities and improve patient care. Due to the powerful ability to mine information and explore the hidden links behind the data, machine learning algorithms have been used in many studies and a wide variety of fields to develop predictive models of disease risk.

As the main caregivers for patients, nurses play a key role in patient condition observation and disease prediction. Compared with traditional risk prediction models or scores, machine learning models are more precise, sensitive, and generalizable, capable of analyzing the deep-seated interaction of multiple factors among data (18) and explore more complex linear or nonlinear correlations. In diverse clinical situations, the capacity to forecast disease risk using the ML technique is greater, which is vital for encouraging medical professionals to intervene early to enhance patient care.

The core of machine learning is the algorithm, which has three main learning patterns: (1) supervised learning, which adjusts the prediction algorithm based on the previous examples to make the prediction results match as close as possible to the output values of the examples when reinput; (2) unsupervised learning, which does not output a value; instead, the training system models the underlying structure of the data; and (3) reinforcement learning, which uses reward/punishment sequences to form strategies for action in a specific problem space through trial and error (19). Machine learning adopts supervised learning algorithms such as support vector machine (SVM), Bayesian learning, decision tree, and regression, and unsupervised learning algorithms such as K-means clustering and association rule learning. Reinforcement learning algorithms (20), such as Q-learning (21) and SARSA, as well as neural networks and other special algorithms, are also implemented in machine learning. At present, the main idea of the quantitative identification technology of disease prediction is to transform the problem of disease risk into a classification problem and then use the corresponding model to perform the classification. According to the literature, the most commonly used and best performing algorithms for disease prediction (22) include SVM, backpropagation (BP) neural network, random forest, and naive Bayes.

However, only single prediction models are implemented in these studies, and the accuracy and stability need to be improved. Ensemble learning is based on the idea of learning from the strengths of others. Constructing and combining multiple machine learning devices to complete the learning task can effectively prevent overfitting and underfitting problems and thus improve the prediction performance (23). In the disease prediction task, there are some problems, such as high feature dimension, multicollinearity between features, and highly noisy physical examination data, that can produce unideal stability in single models. To overcome the above problems and obtain better stability, this paper proposes an ensemble learning method to integrate multiple models to predict disease risk. Bagging and boosting strategies are adopted to evaluate disease prediction based on the ensemble idea.

Prediction models can be coarsely divided into “black-box” and “white-box” models. Most existing prediction models in the medical and health fields are “white-box” models due to the high demands for comprehensibility, interpretability, and transparency. These “white-box” models, which include linear regression and decision tree, have a strong visualization ability but relatively poor prediction precision (24). If the prediction problem is difficult and requires high precision, neural networks, random forests, and other “black-box” models must be used (25).

In recent years, explainable machine learning has become a popular topic in different research fields (26). Explainable machine learning focuses on improving the transparency and credibility of black-box model decision-making. There are two methods for bestowing explicability to a predictive model. First, intrinsically interpretable machine learning methods, such as logistic regression, can be used as the predictive model. Second, postinterpretation methods, such as local interpretable model-agnostic explanations (LIME) (27) and SHapely Additive exPlanations (SHAP) (28), explain complex models through postassisted attribute analysis. This paper improves upon LIME and uses an explainable additive model proposed in recent years to approximate the complex model further to improve the interpretability of the ensemble learning model.

This work aims to overcome the limitations described above by building a COVID-19 diagnostic model based on hematological parameters to provide a new method to screen COVID-19. Different classification models have been developed by applying AI technology to blood test results that can be obtained in a short amount of time (<10 min even in an emergency) and at only a small percentage of the cost of RT–PCR and CT. Our approach can be used to screen COVID-19 patients using regular blood tests in resource-constrained situations, especially during the peak of an outbreak, when RT–PCR reagent shortages become a severe issue. The developed method can also be used as a supplement to RT–PCR tests to increase their sensitivity.

## Methods

### Data Sources

COVID-19 spread rapidly throughout many countries worldwide (29, 30). Early identification of COVID-19 patients and SARS-CoV-2-infected persons is very important and can play a key role in epidemic prevention and control. Therefore, the routine blood test data of patients with COVID-19 was used in this study (31). The data were extracted from a database including the hematochemical values from 1,737 patients (47.00% COVID-19 positive) admitted to San Raphael Hospital (OSR) from February to May 2020. Patient age and sex, the presence of COVID-19-related symptoms at admission (dyspnea, pneumonia, pyrexia, sore throat, influenza, cough, pharyngitis, bronchitis, generalized illness), and a set of hematochemical values from laboratory tests (complete blood count and coagulation, biochemical, blood gas analysis and CO-oximetry values) were considered covariate features. The goal of this study is to classify patients as positive or negative for COVID-19.

### Feature Selection

First, features with no significant differences between the positive and negative COVID-19 groups were eliminated. Student's *t*-test or the Kruskal–Wallis test were used to compare continuous variables, which are presented as the mean ± standard deviation. The chi-square test was used to compare categorical variables, which are presented as frequencies and percentages. A two-tailed *p* value of <0.05 was considered statistically significant. Then, feature correlation analysis was performed according to the Pearson correlation coefficient matrix. Highly correlated features were eliminated to avoid issues related to multicollinearity.

### Machine Learning Algorithms

Four ensemble learning algorithms, including random forest (RF), adaptive boosting (AdaBoost), gradient boosting decision tree (GBDT) and eXtreme gradient boosting (XGBoost), are used as representative boosting algorithms to determine the best performing model. The most optimal variables were further validated using the GBDT method.

Compared with single learning models, the advantage of an ensemble learning model is that it can combine multiple single learning models to obtain more accurate, stable, and robust results (32). The principle of ensemble learning came from the probably approximately correct (PAC) learning model (33). Kearns and Valiant first explored the equivalence of weak and strong learning algorithms (34). Bagging and boosting strategies both combine existing classification algorithms or regression algorithms to form a more powerful predictor. In this paper, RF was used as the representative bagging algorithm. AdaBoost, GBDT, and XGBoost are used as representative boosting algorithms.

#### Bagging

Bagging, also known as bootstrap aggregation, refers to the use of bootstrapping to extract training samples under the same base classifier to train multiple base classifiers and finally obtain the results through a voting method. This approach can help reduce errors caused by random fluctuations in the training data (35). The steps of the bagging process are as follows. The training sets are extracted from the original sample set. In each round, *n* training samples are extracted from the original sample set by bootstrapping, and a total of k rounds of extraction are performed to obtain k training sets. One training set is used to obtain a model, and so k training sets obtain a total of k models. [The model can be determined according to the specific situation; it can be a decision tree, K-nearest neighbor (KNN), etc.] The classification results are produced by voting.

#### Boosting

Boosting transforms weak learners into strong learners through iteration. By increasing the number of iterations, a strong learner with high performance is generated (36); this is considered one of the best performing approaches in machine learning. Boosting increases the weights of samples that were incorrectly classified by the weaker classifier in the previous round and decreases the weights of samples that were correctly classified in the previous round so that the classifier has a better effect on the misclassified data. The final boosting model is obtained according to this rule. The main idea is to combine multiple weak classifiers into one strong classifier. Under the PAC learning framework, the weak classifier must be assembled into a strong classifier.

### Model Validation

All patients were randomly divided into training and testing sets at a ratio of 8:2. To minimize the randomness effect of the training result, 10-fold cross-validation was also adopted. First, the training sets are divided into 10-fold, then the model is trained with nine-fold and verified with the remaining fold. The training is repeated for 10 times, with each a different fold for verification, and the average value of the performance is represented as the generalization performance. Once the models were derived, the performances of the different models were further validated using the receiver operating characteristic (ROC) curve as the evaluation metric. The accuracy, precision, recall, sensitivity, F1 score, youden's index and area under the curve (AUC) were calculated to evaluate the performance of the ML algorithm on testing sets. Finally, the optimal ML algorithm was selected.

### Model Interpretation

The local interpretable model-agnostic explanation (LIME) was used to explain the predictions. The rationale by which a model predicts a single sample using a local linear approximation of the model behavior can be better trusted.

LIME, proposed by (27), is a tool that helps explain how a complex black-box model makes decisions. A new dataset is generated by randomly perturbing the samples in LIME. The new dataset is then used to train a linear model, which locally approximates the black-box model. Then, the local decision behavior of the black-box model is obtained according to the interpretable model.

Note that *x*∈*R*^*d*^ are the samples that need to be interpreted. First, the more important *d*′ dimensional features are selected, and *x* becomes *x*′∈*R*^*d*^′ after removing the less important features. A new sample *z*′ is generated by perturbing *x*′, and the all-new samples constitute a new dataset *Z*′. After adding the removed features to the samples, *z*′ is restored to *Z* ∈ *R*^*d*^. π_*x*_*(z)* is defined as the similarity of samples before and after modification and can be calculated as follows:


(1) $$\begin{array}{r} \pi_{x}\left(z\right)=exp\left(-\frac{{D\left(x,z\right)}^{2}}{\sigma^{2}}\right), \end{array}$$

where *D(x,z)* is the distance formula, whose definition varies with the sample type. When the sample is an image, for example, *D(x,z)* is usually the *L2* norm distance, and when it is text, *D(x,z)* is usually the cosine similarity function.

If f is the complex model to be explained and g is a simple model, the objective function to measure the difference between the two models is as follows:


(2) $$\text{ξ}(\text{x})=\sum_{{\text{z,z}}^{'}}\pi_{\text{x}}(z){\text{f(f(z) - g(z}}^{'}{)}^{2}\;+\Omega (\text{g}),$$

where Ω(g) is the complexity of model g. When g is a linear regression model, the number of nonzero weight coefficients determines the model's complexity. The flow of the LIME algorithm is shown in Table 1.

**Table 1:** Algorithm: LIME.

**Algorithm: LIME**
Input: (1) Complex Model *f*; (2) Samples *X*; (3) Number of randomly generated samples *N*
Steps: 1. Through feature screening, the more important *d*′ features are preliminarily obtained, allowing the interpretation version *X*′ of *X* to be obtained 2. A new sample *Z*′ is generated by randomly perturbing *X*′; then, *Z*′ is restored to *Z* with the same dimensions as *X*. The complex model is used to predict and obtain the labels 3. The newly generated dataset is fitted with a linear model
Output: The weight of the linear model

### Statistical Analyses

Categorical variables were described as number (%) and compared by Chi-square or Fisher's exact test where appropriate. Continuous variables that satisfy normal distribution were described as mean [standard deviation (SD)] and compared by the 2-tailed Student's *t*-test; otherwise, median [interquartile range (IQR)] and Wilcoxon Mann–Whitney *U*-test were used. A two-sided *p*-value <0.05 was considered statistically significant. All statistical analyses were performed with Python (version 3.8.5).

## Results

Among 1,736 patients. 362 patients were excluded because they had more than four missing attribute values. After processing, 1,374 patients remained in the database. Two features (CK and UREA) were removed because their missing value was larger than 30% of their overall value; the average value of each feature was used to fill in the remaining missing values. Thirty-two features were selected for screening patients for COVID-19 (Table 2).

**Table 2:** Characteristics of the positive and negative COVID-19 patients.

	**Total (*N* = 1,374)**	**COVID-19 negative (*N* = 615)**	**COVID-19 positive (*N* = 759)**	***p*-Value**
Age, year	60.40 ± 20.83	60.40 ± 20.83	62.27 ± 15.84	0.066
Female	583 (42.43%)	304 (49.43%)	279 (36.76%)	<0.001
CA, mmol/L	2.20 ± 0.751	2.29 ± 0.74	2.14 ± 0.14	<0.001
CREA, mg/dl	1.18 ± 1.01	1.22 ± 1.20	1.14 ± 0.82	0.180
ALP, U/L	87.74 ± 64.26	94.18 ± 77.16	82.53 ± 50.95	0.001
GGT, U/L	66.12 ± 101.95	58.52 ± 118.90	72.27 ± 85.40	0.013
GLU, mg/dl	119.03 ± 55.85	112.19 ± 49.85	124.58 ± 59.73	<0.001
AST, U/L	47.11 ± 51.37	34.60 ± 33.44	57.25 ± 60.37	<0.001
ALT, U/L	40.15 ± 40.67	32.23 ± 35.22	46.56 ± 43.58	<0.001
LDH, U/L	336.86 ± 210.61	280.76 ± 243.48	382.33 ± 166.44	<0.001
PCR	72.22 ± 79.59	52.86 ± 70.90	89.72 ± 82.43	<0.001
KAL	4.22 ± 0.51	4.25 ± 0.50	4.20 ± 0.52	0.101
NAT	138.58 ± 4.66	139.10 ± 3.92	138.15 ± 5.15	<0.001
WBC, 10^9^/L	8.56 ± 4.75	9.73 ± 5.45	7.62 ± 3.85	<0.001
RBC, 10^12^/L	4.53 ± 0.73	4.40 ± 0.75	4.64 ± 0.69	<0.001
HGB, g/dl	13.18 ± 2.05	12.80 ± 2.13	13.49 ± 1.94	<0.001
HCT, %	39.32 ± 5.64	38.32 ± 5.79	40.14 ± 5.39	<0.001
MCV, fl	87.33 ± 6.93	87.76 ± 7.23	86.97 ± 6.65	<0.001
MCH, pg/cell	29.25 ± 2.69	29.27 ± 2.76	29.23 ± 2.63	0.783
MCHC, g Hb/dl	33.48 ± 1.34	33.34 ± 1.35	33.60 ± 1.32	<0.001
PLT1, 10^9^/L	234.74 ± 95.89	246.55 ± 98.70	225.17 ± 92.51	<0.001
NE, %	72.35 ± 13.26	70.33 ± 13.47	73.98 ± 12.86	<0.001
LY, %	18.58 ± 11.00	19.73 ± 11.37	17.65 ± 10.62	0.001
MO, %	7.83 ± 3.88	8.06 ± 3.61	7.65 ± 4.08	0.045
EO, %	0.88 ± 1.62	1.43 ± 2.02	0.44 ± 1.00	<0.001
BA, %	0.34 ± 0.327	0.43 ± 0.31	0.26 ± 0.21	<0.001
NET, 10^9^/L	6.45 ± 4.48	7.15 ± 5.28	5.88 ± 3.60	<0.001
LYT, 10^9^/L	1.37 ± 0.95	1.64 ± 1.02	1.15 ± 0.83	<0.001
MOT, 10^9^/L	0.62 ± 0.54	0.72 ± 0.45	0.54 ± 0.59	<0.001
EOT, 10^9^/L	0.07 ± 0.14	0.12 ± 0.18	0.03 ± 0.08	<0.001
BAT, 10^9^/L	0.02 ± 0.04	0.03 ± 0.05	0.01 ± 0.02	<0.001
Suspect, %	0.83 ± 0.33	0.71 ± 0.39	0.92 ± 0.23	<0.001

*CA, calcium; CREA, creatinine; ALP, alkaline phosphatase; GGT, gamma-glutamyl transferase, an enzyme that converts glutamyl to glutamine; GLU, glucose; AST, aspartate aminotransferase; ALT, alanine aminotransferase; LDH, lactate dehydrogenase, a type of enzyme that breaks down lactate; WBC, white blood cell; RBC, red blood cell; HGB, hemoglobin, a protein that transports oxygen throughout the body; HCT, hematocrit, a metric representing the proportion of RBCs in the blood; MCV, mean corpuscular volume; MCH, mean corpuscular hemoglobin; MCHC, mean corpuscular hemoglobin concentration; PLT1, platelets; NE, neutrophil count (%); LY, lymphocyte count (%); MO, monocyte count (%); EO, eosinophil count (%);BA, basophil count (%); NET, neutrophil count; LYT, lymphocyte count; MOT, monocyte count; EOT, eosinophil count; BAT, basophil count; Suspect, suspected COVID-19*.

### Baseline Characteristics

Table 2 presents the characteristics of the positive and negative COVID-19 patients. The chi-square test for sex yielded a Pearson's chi-square value of 14.918, and *p* = 0.000 (close to but not equal to zero) <0.05, indicating that the sex differences between the positive and negative COVID-19 groups were significant. In contrast, Student's *t*-test or the Kruskal–Wallis test showed that there was no difference in age, CREA, KAL, or MCH between the two groups (*p* > 0.05).

**Figure 1:**
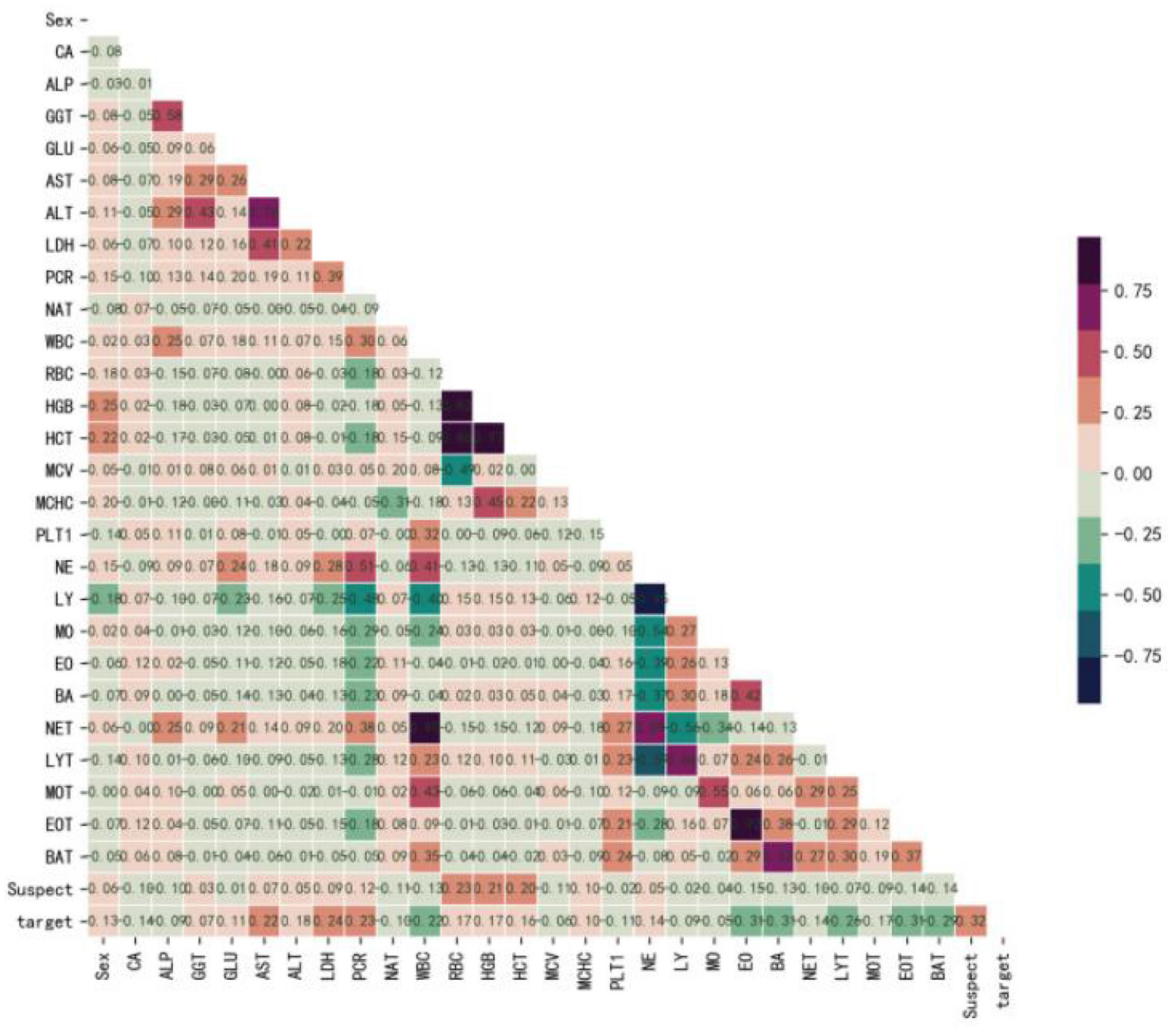
Correlation coefficient matrix heatmap of all 29 variables. The obtained numerical matrix is visually displayed through a heatmap. Orange indicates a positive correlation, and green indicates a negative correlation. Color depth indicates the value of the coefficient, with deeper colors indicating stronger correlations. Specifically, redder colors indicate correlation coefficients closer to 1, and greener colors indicate coefficients closer to −1.

Figure 1 shows that Sex (*r* = 0.13), GGT (*r* = 0.07), GLU (*r* = 0.11), AST (*r* = 0.22), ALT (*r* = 0.18), LDH (*r* = 0.24), PCR (*r* = 0.23), RBC (*r* = 0.17), HGB (*r* = 0.17), HCT (*r* = 0.16), MCHC (*r* = 0.10), NE (*r* = 0.14), and Suspect (*r* = 0.32) were positively correlated with the target, while, CA (*r* = −0.14), ALP (*r* = −0.09), NAT (*r* = −0.10), WBC (*r* = −0.22), MCV (*r* = −0.06), PLT1 (*r* = −0.11), LY (*r* = −0.09), MO (*r* = −0.05), EO (*r* = −0.31), BA (*r* = −0.31), NET (*r* = −0.14), LYT (*r* = −0.26), MOT (*r* = −0.17), EOT (*r* = −0.31), and BAT (*r* = −0.29) were negatively correlated with the target. Therefore, we believed that there were no redundant features and selected all of them to develop the model.

### ML Algorithms' Performance Comparison

Data from 80% of the 1,374 patients were randomly selected and used as the training set, while the data from the remaining 20% of the patients were used as the testing set. The prediction models were developed with the training set, and their performance was evaluated with the testing set. Random forest, AdaBoost, GBDT, and XGBoost were selected as the typical algorithms of the ensemble learning model. The performance of the ML models was evaluated by using the area under the receiver operating characteristic curve (AUC).

The GBDT algorithm had the best fitting effect on the COVID-19 dataset, with an accuracy of 93.8% and an AUC of 98.4% [95% CI (0.978, 0.990)] on the training set and 80.4 and 86.4% [95% CI (0.821, 0.907)], respectively, on the test set (see Tables 3, 4 for details on the performance metrics).

**Table 3:** Performance of random forest, AdaBoost, GBDT, and XGBoost models in screening COVID-19.

**Model**	**Accuracy**	**Precision**	**Recall**	**Sensitivity**	**F1 score**	**Youden's index**
**Random forest**	74.2%	70.8%	90.8%	53.7%	0.795	0.589
**AdaBoost**	76.7%	78.2%	80.3%	72.4%	0.792	0.553
**GBDT**	80.4%	80.3%	85.5%	74.0%	0.828	0.615
**XGBoost**	75.3%	73.3%	86.8%	61.0%	0.795	0.565

**Table 4:** Performance of random forest, AdaBoost, GBDT, and XGBoost models to screen COVID-19.

**Model**	**AUC**	**AUC_95% CI**	**AUC_SD**	**AUC_*p* value**	**Confusion matrix**
**Random Forest**	85.7%	0.813, 0.902	0.02	<0.001	[66, 57], [14, 138]
**AdaBoost**	85.4%	0.810, 0.899	0.02	<0.001	[89, 34], [30, 122]
**GBDT**	86.4%	0.821, 0.907	0.02	<0.001	[91, 32], [22, 130]
**XGBoost**	84.9%	0.803, 0.894	0.02	<0.001	[75, 48], [20, 132]

**Figure 2:**
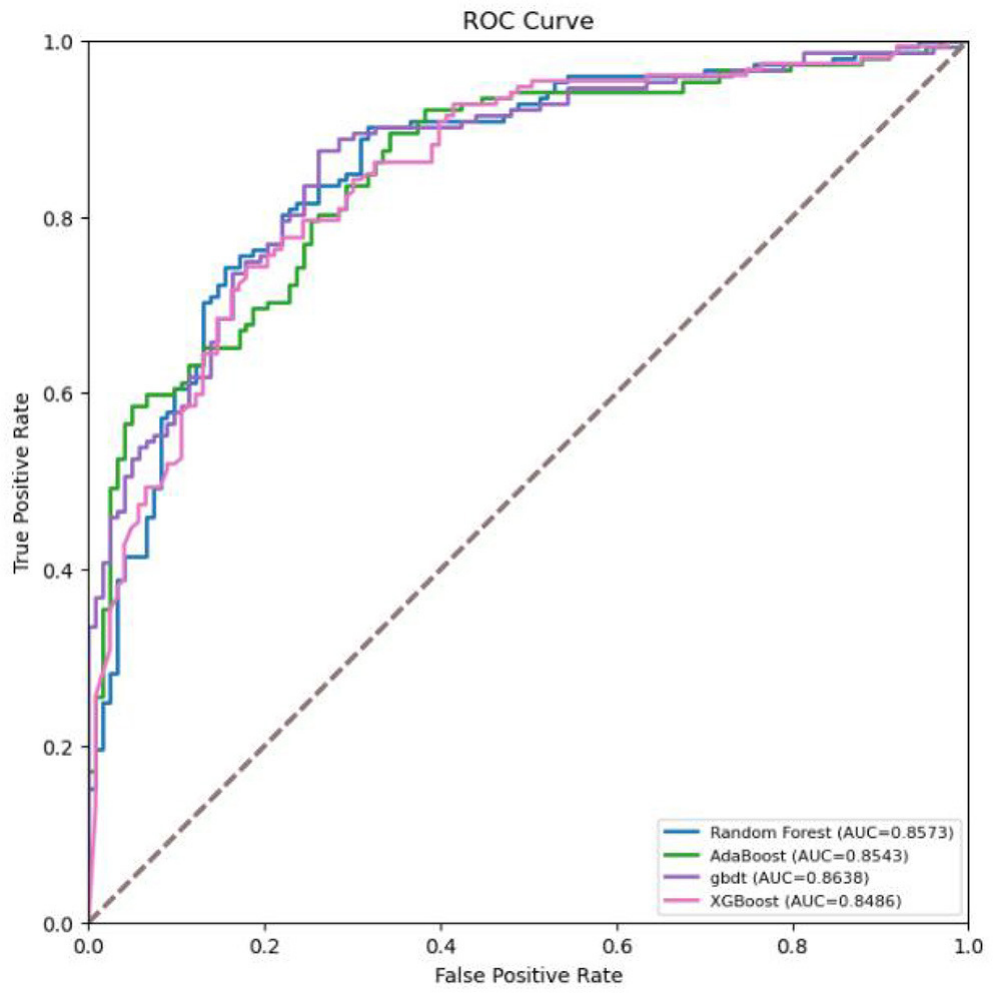
Receiver operating characteristic (ROC) curves for the machine learning models in screening COVID-19.

As shown in Figure 2, the performance of GBDT was better than that of random forest, AdaBoost, and XGBoost. DeLong's test was further used to assess the difference between two AUCs, which confirmed that the AUC of the GBDT model was significantly different from that of the other three models (*p* < 0.01).

**Figure 3:**
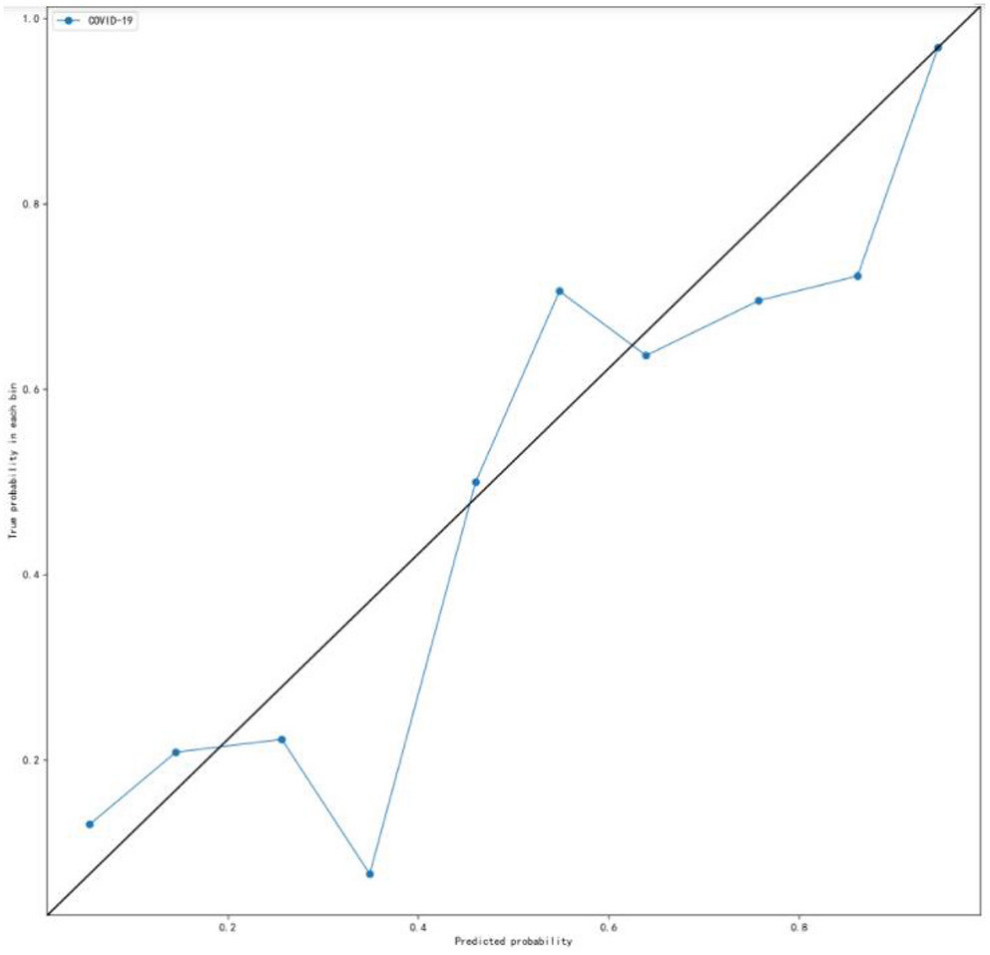
Calibration curve for the internal validation set. The calibration curve was plotted using the bucket method (continuous data discretization) to observe whether the prediction probability of the classification model is close to the empirical probability (that is, the real probability). Ideally, the calibration curve lies along the diagonal (i.e., the prediction probability is equal to the empirical probability).

A calibration curve was obtained with the bucket method (continuous data discretization) to observe whether the prediction probability of the classification model was close to the actual probability. It is an evaluation index of a probability model. The calibration curve of the GBDT model was drawn with the predicted probability as the abscissa and the true probability in each bin as the ordinate. As shown in Figure 3, the calibration curve was close to the diagonal, indicating that in the model testing experiment, the GBDT model performed well.

### Explanation of the Best Model

#### Feature Importance of GBDT

**Figure 4:**
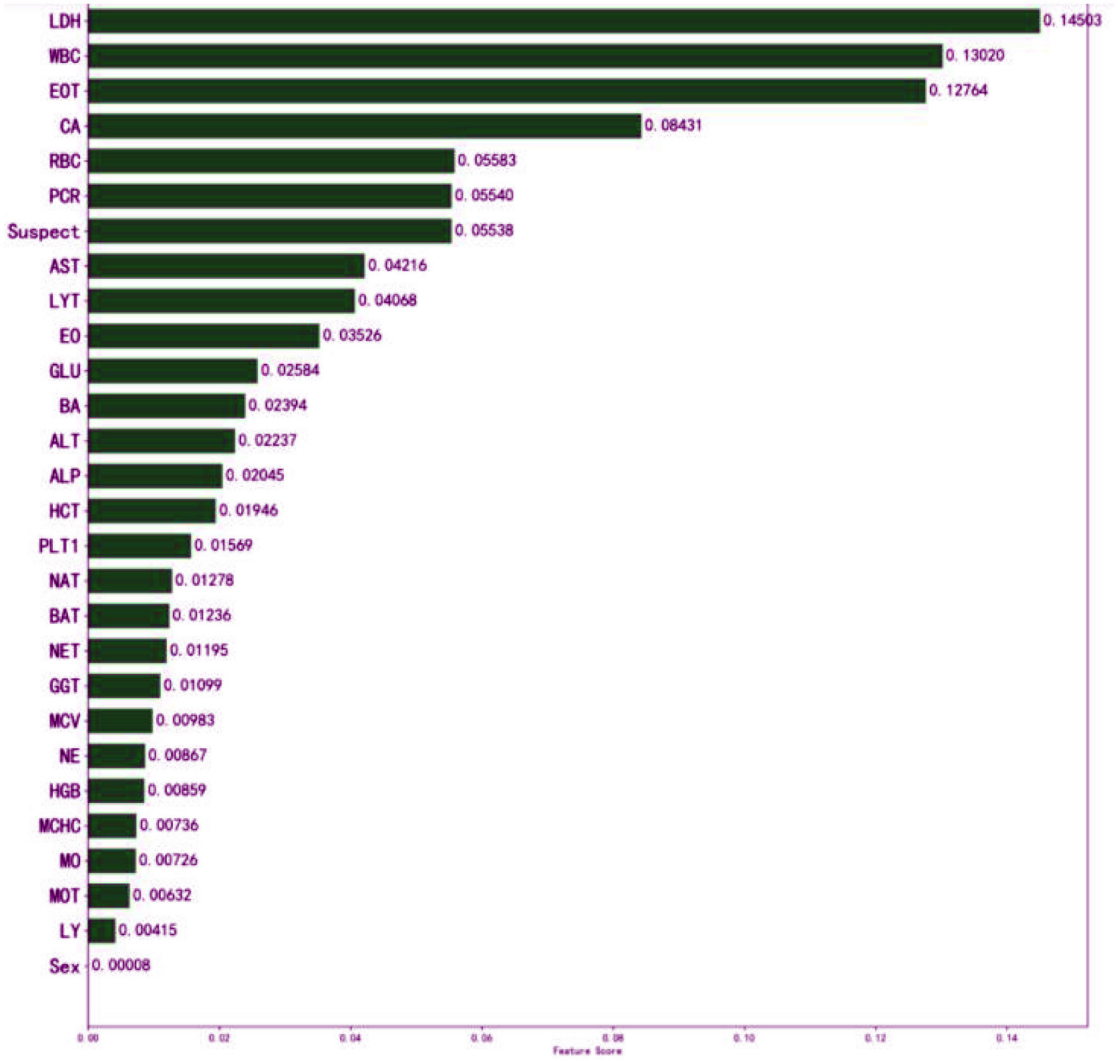
Influence of input features on the outcome of the XGBoost model. The top three features are LDH, WBC, and EOT. It indicates that they have important auxiliary diagnostic significance for COVID-19. The model found that patients with higher WBC count, higher LDH level, or higher EOT count, were more likely to have COVID-19. It might assist physicians to make their decisions.

The meaning of “GradientBoostingClassifier (*n*_estimators = 100, learning_rate = 1.0, max_depth = 1, random_state = 0)” in classifying the patients could not be explained to the doctors sufficiently. In general, the interpretability of GBDT is reflected in its feature importance. The feature importance derived from the XGBoost model is shown in Figure 4.

#### Interpretation by LIME

**Figure 5:**
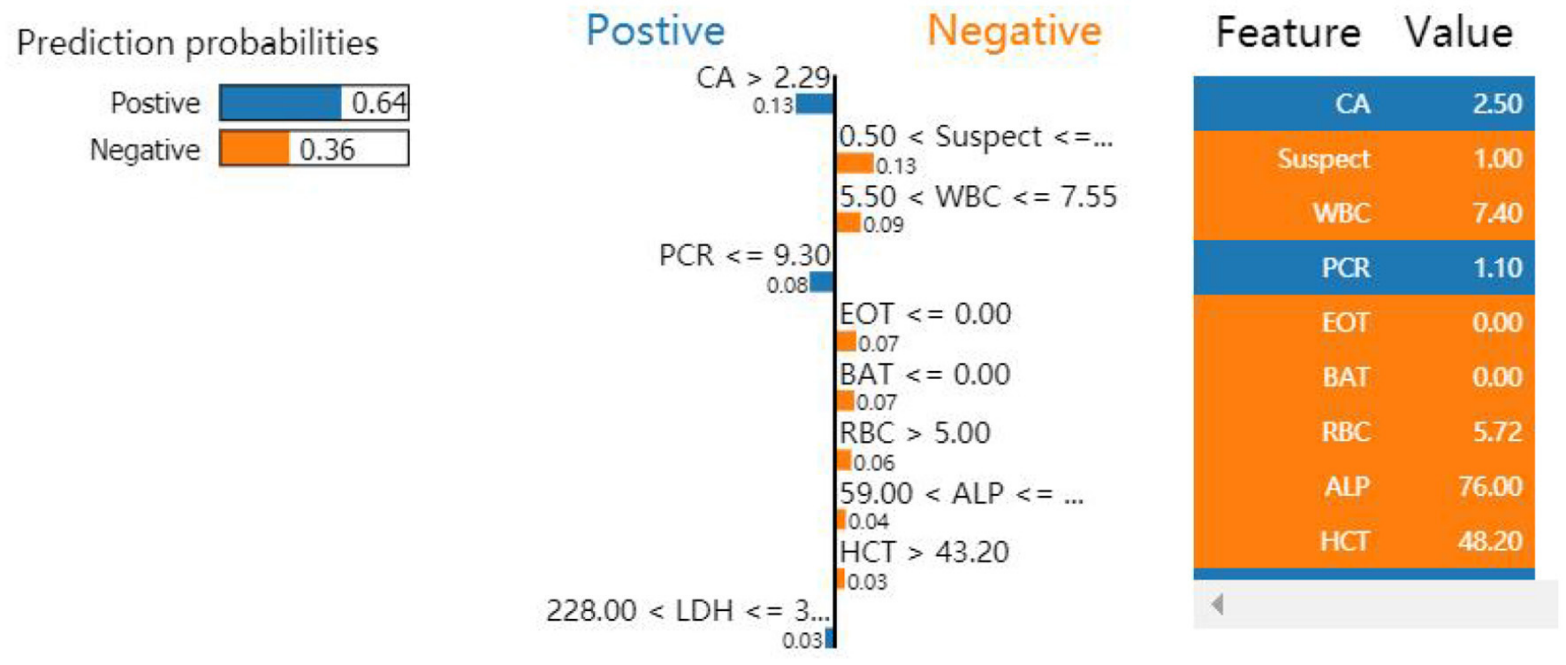
Influence of nine variables on the outcome of the XGBoost model. Because PCR ≤ 9.30 and CA >2.29 were the most significant features, the classification of this sample was confirmed as positive.

**Figure 6:**
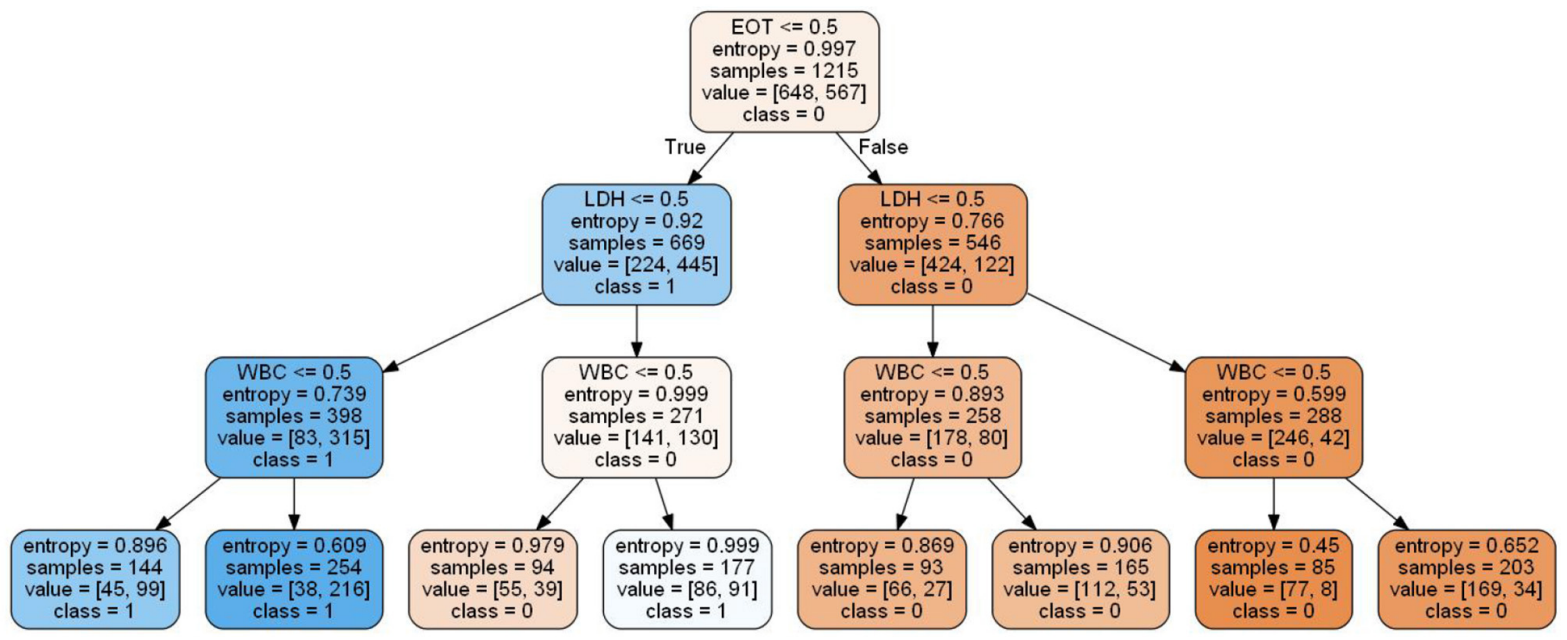
Simplified decision tree model based on the top three features.

Local interpretable model-agnostic explanations selects a specific sample in the test dataset to obtain the probability value of each class and explains the reason for assigning the probability. Figure 5 shows the prediction results of the sample. The figure shows which features determined that the sample should be classified as COVID-19 positive (blue) and which determined that the sample should be classified as COVID-19 negative (orange). The values of the features for the sample are listed in the figure to show the contribution of the features. Specifically, CA, PCR, and LDH were important factors for determining positive COVID-19 patients. These three features were further discretized and used to developed a simplified decision tree model (Figure 6).

## Discussion

The COVID-19 outbreak is currently under control in China and is in a state of normalized prevention and control, but imported cases from other countries occur often, and the number of infections worldwide continues to rise. Virus nucleic acid detection is the “gold standard” for the diagnosis of COVID-19. However, due to premature collection times, nonstandard collection methods, and inaccurate collection locations, false negative results have occurred many times in virus nucleic acid detection (37). Chest CT plays an important role in the early diagnosis of COVID-19, with a high sensitivity but low specificity (25%) (38). Therefore, developing a new strategy for achieving a rapid and accurate diagnosis for COVID-19 is essential from a clinical perspective.

Since the start of the COVID-19 outbreak, a large number of scholars have been committed to applying AI technology to rapidly diagnose COVID-19. Wu et al. (39) constructed a COVID-19 differential diagnosis model by mining 11 key blood indices through an ML algorithm and obtained accuracy rates of 0.9795, 0.9697 and 0.9595 with their cross-validation set, test set and external validation set, respectively. Li et al. (40) developed a deep learning model based on CT images to distinguish COVID-19 from community-acquired pneumonia. With the independent validation set, the AUC for identifying COVID-19 was 0.96 and that for identifying community-acquired pneumonia was 0.95. Ozturk et al. (8) constructed a deep learning classification model based on the chest X-ray films of COVID-19 patients. The results showed that the accuracy of the model in performing two-class and multiclass classification were 0.9808 and 0.8702, respectively. All the AI models in the above studies showed good diagnostic performance but only included a single index for evaluation and analysis and participation in model construction (laboratory examination index or chest image index). Combined with the comprehensive analysis of clinical manifestations, laboratory examination, CT and other indicators, this study jointly constructed a predictive diagnosis model for COVID-19 based on ML that better reflects the real-world COVID-19 situation.

Artificial intelligence technology has an excellent ability to process big data and mine complex medical information. In medical scenarios, the most common problem is binary classification, such as predicting whether a patient has a disease through data analysis and model establishment. Simple models used to solve classification problems include logistic regression, decision tree, and SVM. However, due to the limitations of these simple models, they often cannot achieve optimal prediction efficiency, so the application of ensemble learning models is becoming more widespread in the machine learning field. AdaBoost was the first boosting model and functions by training different weak models based on the same training dataset and then integrating these weak models to form a stronger classifier with a better effect. XGBoost is a machine learning method focusing on the gradient lifting algorithm. The loss function is expanded as a second-order Taylor expansion, the second derivative of the loss function is used to optimize the loss function, and depending on whether the loss function is reduced, a decision on whether to split nodes is made. The disadvantage of XGBoost is that it is sensitive to outliers.

In GBDT, a tree is trained first by using the training set and the real classification of the samples; then, the tree is used to predict the classification of the training set to obtain the predicted value of each sample, and the deviation between the predicted value and the true value, that is, the residual, is used as the standard answer to train the next tree. Then, the residual is used to train a third tree, and the final prediction result is obtained. Because the growth process of the decision tree continuously selects and segments features, GBDT composed of a large number of decision trees has inherent advantages and can easily yield the importance ranking of its features. The advantages of the chosen methods over the others are as follows. (1) The prediction accuracy is higher, it is more suitable for low-dimensional data, and it can contend with nonlinear data. (2) It can flexibly handle various types of data, including continuously and discretely valued data. (3) In the case of a relatively short parameter adjustment time, the preparation rate of the prediction can be high relative to that of SVM. (4) Certain robust loss functions, such as the Huber and quantile loss functions, make the model very robust to outliers.

The model constructed in this study has high clinical application value. The three features identified, LDH, WBC, and EOT, can assist doctors in rapidly and accurately diagnosing COVID-19 patients. Under normal circumstances, LDH is limited to the cytoplasm of tissue cells; it is released only when cell damage and necrosis cause an increase in cell membrane permeability, resulting in a rise in serum LDH concentration. The degree of lung tissue injury is directly proportional to the level of serum LDH, so the level of serum LDH can indirectly reflect the severity of the disease. The sickness is mild when a patient is first infected with SARS-CoV-2. As the disease progresses, the condition gradually worsens, and the LDH level gradually increases (41, 42). The number of white blood cells in a unit volume of blood is measured by the white blood cell count (WBC). White blood cells are an important part of the body's defense system and a common marker for identifying infection, with a high specificity in the diagnosis of infectious fever. According to previous research, infection should still be considered first when the WBC rises. SARS-CoV-2 infection stimulates the innate and adaptive immune responses of the infected body, resulting in a series of inflammatory reactions and pathological changes. The excessive immunological response of the body to external stimuli such as viruses and bacteria is referred to as a cytokine storm (43). It can cause the body to quickly produce a large number of cytokines, such as IL-6, IL-12, IL-8, and IFN-α; this abnormal increase in the number of cytokines can cause aggregation of eosinophils and other infectious lesions. The organs and tissues are also severely damaged in the process of effectively eradicating the infection (44, 45).

The application of AI technology in the medical field has created new opportunities for solving many medical challenges. However, it can be difficult for users to understand the internal working principle and decision-making process of the model due to its inherent inexplicability. This reduces doctors' trust and acceptance of the AI model and limits the development of AI products in the medical field. Therefore, the construction of interpretable AI models has become the focus of research in recent years. The decision tree model can reflect both linear and nonlinear relationships, allowing it not only to make accurate predictions but also to be interpretable (46). The interpretability of the model is reflected in both global interpretability and local interpretability. The global interpretability shows that the decision tree model can visualize the weight of each index variable, allowing it to assess the value of each index in the prediction model. The higher the index weight value is, the greater the importance of the index. In this study, LDH was the most important index in the construction of the GBDT model, with a weight value of 0.145. Local interpretability explains the diagnosis results for a specific case, which can indicate which indicators support the diagnosis of the disease, which indicators deny the diagnosis of the disease, and the basis for the diagnosis, which is helpful in making an individualized prediction for each patient and providing accurate treatment. To determine whether a patient is infected with COVID-19, the patient is selected from the validation set and input into the LIME model. The results show that although the CA and PCR2 indicators confirm that the model can diagnose COVID-19 patients, all other indicators deny a diagnosis of COVID-19; the overall tendency, however, is toward a positive diagnosis of COVID-19 for the patient, consistent with the actual patient diagnosis (Figure 5).

In the fight against COVID-19, top international journals have published many research results, including epidemiological and clinical feature analysis, epidemic trend prediction, death-related risk factors, prognostic impact of basic diseases, and critical disease prediction models, which provide important scientific support for this fight and play a positive role in guiding epidemic prevention and control. In a study published in the Lancet, a susceptible-exposed-infectious-recovered metapopulation model was used to simulate epidemics across all major cities in China. The study suggested that preparedness plans and mitigation interventions should be readied for quick deployment globally (47). In a study published in JAMA, Pan et al. (48) applied surveillance data to quantify the temporal evolution of the intensity of COVID-19 transmission across different periods. Their study may have important implications for ongoing and potential future nonpharmaceutical bundles in the US and other nations with respect to daycare for children (49). Liang et al. (50) developed a clinical risk score to predict the occurrence of critical illness in hospitalized patients. The score may help identify patients with COVID-19 who may subsequently develop a critical illness. Vaid et al. (51) developed machine learning models to predict critical illness and mortality in a cohort of patients in New York City. These models identified at-risk patients and uncovered underlying relationships that predicted patient outcomes. In most studies, a kind of model was applied without considering the ensemble learning algorithms.

This study used a small sample of COVID-19 patients, which may affect the accuracy of the results. Additionally, utilizing a deep learning model with such a small sample size is not ideal. The dataset is not sufficiently standardized, resulting in the elimination of several indicators due to the large number of missing values. In future research, the sample size must be further increased, and a more standardized sample set should be selected to confirm the results of this study.

## Conclusions

In this study, random forest, AdaBoost, GBDT, and XGBoost algorithms were used to develop bagging and boosting ensemble learning models to predict disease risk and then compared in terms of the AUC, accuracy, recall, and F score. Finally, the optimal model was explained by way of the LIME algorithm. Taking the COVID-19 data as a case study, the research is summarized as follows.

First, compared with other classifiers, the precision of GBDT was 80.3%, and the recall was 85.6%. The AUC was 86.4% [95% CI (0.821, 0.907)], indicating better performance. Therefore, GBDT was chosen as the prediction model for the early diagnosis of COVID-19. The model, which was developed based on blood tests, can provide an alternative method to rRT-PCR for the fast and cost-effective identification of COVID-19-positive patients. It is especially effective in places where outbreaks are on the rise.

Second, the risk factors in the prediction model were visualized using the LIME algorithm. CA, PCR, and LDH were revealed as important factors for identifying patients positive for COVID-19. These findings can help doctors control and treat patients in a timely manner. In addition, the same method can be extended to predict other diseases.

Third, in future studies, multiple features will be fused to enhance the richness and effectiveness of the features. In the ensemble strategy, stacking is a hierarchical model integration framework that will be incorporated into an integration model in future studies. Finally, for classification algorithms, the most popular models were tested. To obtain improved precision in early disease risk identification, combinations of models will be investigated, model complexity will be reduced, and graph neural networks will be integrated in future works.

## Data Availability Statement

The original contributions presented in the study are included in the article/supplementary material, further inquiries can be directed to the corresponding author.

## Ethics Statement

Ethical review and approval was not required for the study on human participants in accordance with the local legislation and institutional requirements. Written informed consent from the participants' legal guardian/next of kin was not required to participate in this study in accordance with the national legislation and the institutional requirements.

## Author Contributions

Methodology, software, validation, and visualization: HG. Data curation: MW. Writing—original draft preparation: HZ, ME, and HG. Writing—review and editing: HZ, ME, and MW. Supervision: MW and MJ. Project administration: HG and MJ. All authors have read and agreed to the published version of the manuscript.

## Funding

This research was funded by the Natural Sciences Foundation of Hunan Province (Grant No. 2021JJ30139), the National Natural Science Foundation of China (Grant No. 61773157), and the Key Project of R & D plan of Changsha (Grant No. kq2004011).

## Conflict of Interest

The authors declare that the research was conducted in the absence of any commercial or financial relationships that could be construed as a potential conflict of interest.

## Publisher's Note

All claims expressed in this article are solely those of the authors and do not necessarily represent those of their affiliated organizations, or those of the publisher, the editors and the reviewers. Any product that may be evaluated in this article, or claim that may be made by its manufacturer, is not guaranteed or endorsed by the publisher.

## Abbreviations

AdaBoost: adaptive boosting
AUC: area under the curve
BP: backpropagation
CBC: complete blood count
CI: confidence interval
GBDT: gradient boosting decision tree
GGT: gamma-glutamyl transferases
HCT: hematocrit
HGB: hemoglobin
JMIR: Journal of Medical Internet Research
LDH: lactate dehydrogenase
LIME: local interpretable model-agnostic explanations
ML: machining learning
OSR: San Raphael Hospital
PAC: probably approximately correct
RF: random forest
RCT: randomized controlled trial
XAI: explainable artificial intelligence
XGBoost: extreme gradient boosting.
